# Surgical intervention in patients with proximal femoral fractures confirmed positive for COVID-19—a report of 2 cases

**DOI:** 10.1080/17453674.2020.1770420

**Published:** 2020-06-05

**Authors:** Suk Kyoon Song, Won Kee Choi, Myung Rae Cho

**Affiliations:** Daegu Catholic University Medical Center, Daegu, South Korea

Demographics, clinical characteristics, and laboratory findings of the two patients

Case 1 (Figure 1): An 81-year-old woman presented to a local clinic because of hip pain after a fall on March 17, 2020. Her chest radiographs revealed pneumonia and she was referred to our hospital. At our emergency department, a diagnosis of COVID-19 was confirmed by chest imaging studies and positive viral nucleic acid tests on throat swab specimens (Hong et al. 2020). The patient used a cane for walking and had diabetes mellitus (DM), chronic kidney disease (CKD), chronic obstructive pulmonary disease (COPD), atrial fibrillation, and was on apixaban. A pacemaker was implanted in 2007 to treat sick sinus syndrome, and she had undergone mitral valve replacement surgery because of mitral valve stenosis. Plain radiographs of the pelvis showed an intertrochanteric fracture (AO 31A2.2) of the right femur (Müller et al. 2012). On presentation, there were no COVID-19 symptoms, such as fever, cough, or fatigue. She also showed no dyspnea on room air. She was admitted to the COVID-19 isolation ward, and antibiotic treatment (lopinavir/ritonavir twice daily, levofloxacin 750 mg IV every 24 hours, ceftriaxone 2 g IV every 24 hours) was initiated. During the admission her symptoms did not worsen, and she did not require oxygen therapy. She had to omit apixaban for 2 days in order to reduce bleeding risk. We were able to perform surgery within 72 hours of admission. Due to poor visibility from wearing protective devices, a videoscope was used for endotracheal intubation. The patient was put under general anesthesia and placed in the supine position on a fracture table. After closed reduction under fluoroscopic guidance, we used the direct lateral approach to the hip and fixated the fracture with a compression hip screw and a trochanteric stabilizing plate (Solco, Pyeongtaek, Korea). The operative time (from skin incision to the end of skin closure) was 45 minutes. Since droplets frequently occur during extubation, only an anesthesiologist and a nurse anesthetist remained in the operating room during extubation after surgery. Blood loss during surgery was estimated to be 450 milliliters. From the day after surgery, the patient was encouraged to ambulate with a walker. Antibiotic treatment was continued during her stay on the isolation ward. She showed no symptoms of COVID-19 and there was no worsening of chest radiograph findings at 2 weeks after the operation.

Case 2 (Figure 2): An 83-year-old woman presented to a local clinic with dyspnea on March 20, 2020. Her chest radiograph showed no active pulmonary lesions but she was confirmed positive for COVID-19 by viral nucleic acid tests on throat swab specimens. She resided in a nursing-home facility before presentation to the local clinic and had hypertension and dementia. On March 31, while in the isolation ward in the local clinic, she fell off of the bed. Plain radiographs of the pelvis showed a transcervical fracture (Garden type 4) of the left femoral neck. She underwent intramedullary nailing surgery of the ipsilateral femur 3 years prior due to a femoral shaft fracture.

She was referred to our hospital for surgical care. No symptoms, such as fever, cough, or fatigue, were seen on presentation. She also showed no dyspnea on room air, and there were no radiographic findings of pneumonia on chest radiographs. She was admitted to the COVID-19 isolation ward, and antibiotic treatment (hydroxyquinoline 400 mg PO daily, ceftriaxone 2 g IV every 24 hours) was initiated. During admission, her symptoms did not worsen and she did not require oxygen therapy. We were able to perform surgical treatment within 43 hours of admission. The patient underwent surgery while wearing a Korean Filter 94 (KF 94) facemask and with supplemental oxygen of 2 liters per minute via nasal cannula. Under spinal anesthesia, the patient was positioned laterally, and the intramedullary nail was removed initially. We used the modified Hardinge approach and performed a bipolar hemiarthroplasty. A crack occurred in the greater trochanteric region during removal of the intramedullary nail, and was fixated with 2 cannulated screws, 18-gauge wire, and a multifilament cable. The operative time (from skin incision to the end of skin closure) was 59 minutes. Blood loss during surgery was estimated to be 600 milliliters. From the day after surgery, the patient was encouraged to ambulate with a walker. Antibiotic treatment was continued during her stay on the isolation ward. She showed no symptoms of COVID-19 and there was no worsening of chest radiograph findings at 2 weeks after the operation.

## Discussion

Over 1.3 million people from 213 countries had been confirmed positive for COVID-19 by April 7, 2020, and 73,497 related deaths occurred (6% case fatality rate [CFR]). While the CFR varies by country and by patient characteristics, CFR generally increases with age. According to a study by the Chinese Center for Disease Control and Prevention, the CFR was: age 40–49, 0.4%; 50–59, 1.3%; 60–69, 3.6%; 70–79, 8.0%; and 80 or older, 14.8% (Onder et al. 2020). During this pandemic, as millions of patients are testing positive for COVID-19, the diagnosis and treatment of conditions other than COVID-19 may be delayed, causing additional morbidity and mortality. Patients with proximal femoral fractures are mostly elderly and many have several comorbidities. Overall mortality rate following proximal femoral fracture is above 25%, and the rate is higher when surgical intervention is delayed. If a patient with proximal femoral fracture is confirmed positive for COVID-19, the response to COVID-19 may be prioritized and management of the fracture may be delayed. Clinically, COVID-19 has a reported incubation period of approximately 5–6 days, and symptoms last for about 2 weeks on average (Onder et al. 2020). However, the clinical course may vary in different patient populations, and it may take several weeks for some patients to be completely cured. Even though proximal femoral fracture is not an emergency condition, early surgical intervention contributes to better prognosis and puts the patient at lower risk of mortality. If proximal femoral fracture surgery is delayed for too long, complications of the fracture, rather than the COVID-19 itself, may put the patient at greater risk of morbidity (Moja et al. 2012, Vrahas and Sax 2017, Seong et al. 2020). Thus, this decision needs to be elaborated considering the clinical condition of the patient. For example, if the patient suffers from severe pneumonia due to COVID-19, it may be safer to delay the surgery until after the pneumonia resolves. However, in patients with mild COVID-19-related pneumonia, if the timing of surgery is delayed, complications of the fracture, rather than COVID-19, may put the patients at a greater risk for mortality. Therefore, early surgical intervention for fractures may contribute to better prognosis. Of our 2 patients, 1 patient developed mild pneumonia, while the other did not show radiographic features or signs of pneumonia. The patients were clinically stable after surgery, with no complications.

All surgical procedures were conducted in a negative-pressure operating room dedicated to patients with infectious diseases, and located in a remote corner of the operating complex, with separate access (Ti et al. 2020). All equipment for surgery and anesthesia was prepared and covered with sterile drapes in advance of the patient entering the operating room. Biological isolation chambers with negative-pressure filtration systems were used to move the patients from the isolation ward to the operating room. Patients wore KF 94 face masks throughout the entire surgical procedure, except when they were intubated under general anesthesia. KF 94 is an abbreviation for “Korean Filter 94.” The number 94 indicates a 94% ability to protect against fine particles 0.4 µm in size. It blocks the passage of fine droplets of saliva or sputum from an unpredictable cough.

The traffic in an operating room is a risk factor for increased postoperative infection and exposure to COVID-19. We attempted to minimize the number of staff members in the operating room at any one time. Only 6 staff members (4 surgeons and 2 nurses) attended the operation. An experienced surgeon is important to decrease surgical time and potential preoperative complications. The surgeon who operated on these patients was an expert in hip surgery, who had performed more than 100 proximal femoral fracture surgeries annually since 2004. All surgeons and scrub nurses donned level D personal protective equipment (PPE) with N-95 facemasks (Forrester et al. 2020) (Figure 2F) and the anesthesiologist and nurse anesthetist donned level D PPE with N-95 facemasks and powered air-purifying respirators (PAPR). Surgeons and scrub nurses cannot use powered air-purifying respirators because the equipment can prevent the wearing of a sterile operation gown and restrict the surgeon’s activity.

**Figure 1.**
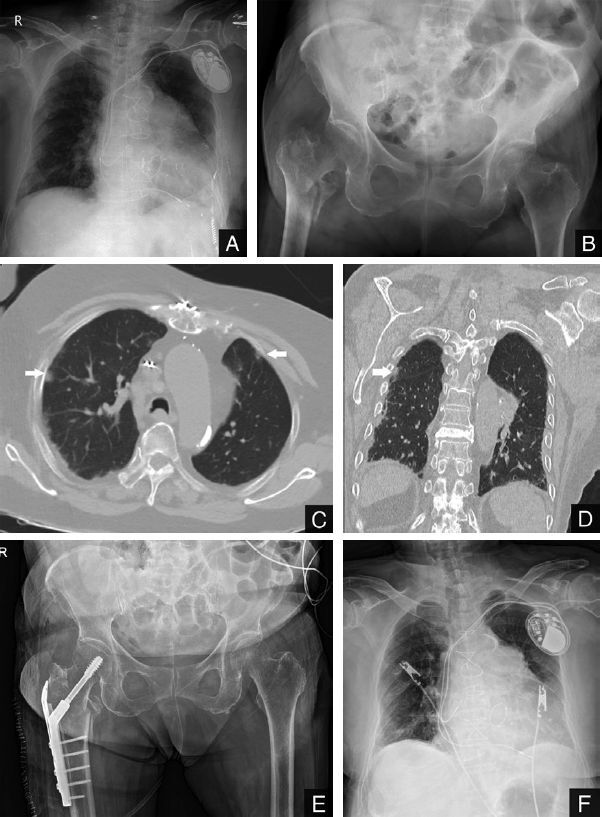
Radiographic images of the chest and the hip. Plain radiographs (Panels A and B) obtained at our emergency department show prominently increased interstitial opacities at the peripheral area in both lungs (A) and right femur intertrochanteric fracture (B). Axial and coronal CT images (Panels C and D) show nodular opacities based in the subpleural area (arrows) and a small amount of bilateral pleural effusion. Panels E and F are plain radiographs taken the day after surgery.

**Figure 2.**
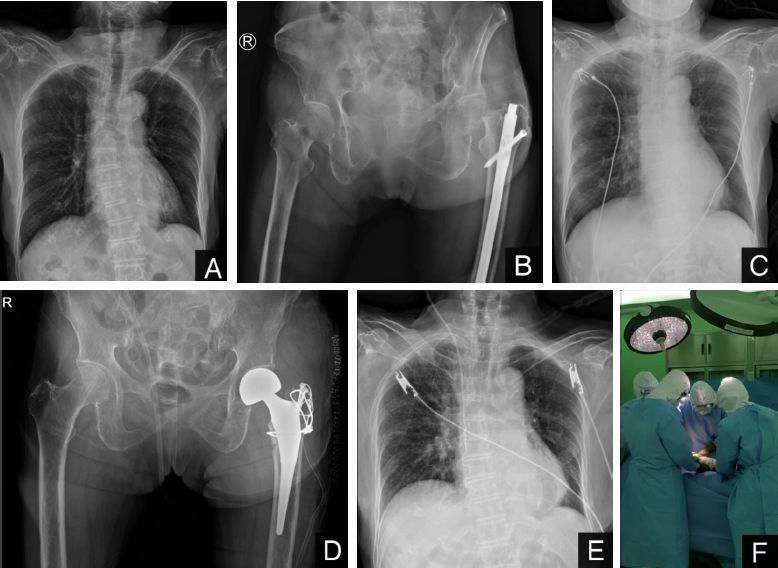
Chest radiographs (Panels A and C) obtained in a local clinic (A) and after presentation to our hospital (C) showing no active pulmonary lesions. Panel B shows a left femur neck fracture. Panels D and E are plain radiographs taken the day after surgery. Panel F shows the surgical team performing surgery while wearing level D PPE.

The first patient was intubated because of the prolongation of prothrombin time from the use of apixaban due to atrial fibrillation. The prolongation of coagulation time is a contraindication for spinal anesthesia because damage to the epidural vein during spinal anesthesia may trigger epidural hematoma and cause spinal cord compression (Olawin and Das 2019). We chose the surgical approach in the usual manner. Since COVID-19 patients are more prone to developing pulmonary symptoms, we used a tapered non-cemented stem (Zimmer, Warsaw, IN, USA) in Case 2 as it can reduce surgical time and has been associated with a lower incidence of pulmonary embolism than the cemented type.

Existence of viral particles in the blood is still a concern. Therefore, the use of power tools such as bone saws and bone drills during surgery might accompany a risk of transmitting viral particles in body fluids and tissues; SARS-CoV-2 is known to be present in all bodily fluids. We also used bulb syringes rather than pulsatile irrigation to minimize the spraying of blood and bodily fluids. Electrocautery for cutting and coagulation may increase the risk of infection via aerosol generation. In order to minimize exposure to aerosols we lowered the power settings used for electrocautery and minimized its use. Electrocautery smoke was evacuated exhaustively using a suction device.

Surgical staff members were kept out of the operating room during the intubation and extubation procedures in order to minimize transmission via droplets from unpredictable coughing; they stood by in a sterile corridor at the ready for an emergency situation. The routine duration of postoperative antibiotic use in our orthopedic department is 72 hours. Since COVID-19 patients are more prone to developing viral pneumonitis, preventive antibiotics were used to prevent bacterial super-infection in these at-risk patients. Combination antiviral therapy should be added in patients with comorbidities and elderly patients (aged over 65 years).

Both patients had a considerably long waiting time before surgery. The procedures for diagnosing COVID-19 and preoperative cardiopulmonary risk assessment were the main causes of delay. In particular, the first patient had to halt apixaban for 2 days in order to reduce bleeding risk. Apixaban is recommended to be discontinued 48 hours before high-bleeding-risk procedures such as major orthopedic surgery (Mandernach et al. 2015). (We discussed this matter with the department of cardiology.) The second patient initially was admitted to a local clinic for other causes and later referred to our hospital after the fracture event. It took several days to assess comorbidities and preoperative risks. We would have postponed surgery if the risks of COVID-19 were more severe than the risks of surgical delay (e.g., severe or bilateral pneumonia, high oxygen demand).

The still-unknown characteristics of the disease and the high mortality rate in patients with risk factors may cause fear in healthcare workers managing patients confirmed positive for COVID-19. Medical personnel must approach such situations cautiously until vaccines or therapeutic agents for COVID-19 are developed. More caution is necessary for patients who are in higher risk categories. However, as in our study, in COVID-19-positive patients with no or mild symptoms, early surgical intervention for comorbid conditions may be carried out if active precautions are taken. If protective measures are taken, doctors and nurses may safely perform surgery, especially because compared with SARS or MERS, which were more often fatal, COVID-19 is reported to show a lower fatality (Lin et al. 2020, Wang et al. 2020).

We followed the treatment guidelines of our hospital at all times when managing these 2 patients. There was no exacerbation of COVID-19 symptoms or radiographic findings; it is unclear whether this fact is attributable to antiviral therapy, or whether it is because COVID-19 is in many cases a self-limiting disease that requires only supportive care. This remains a limitation of our study, and further research is needed to decide the matter. Neither of the participants displayed any symptoms of COVID-19 in the observation period, which lasted 14 days after surgery.

In conclusion, based on the fact that early surgical intervention for proximal femoral fractures in elderly patients may result in superior surgical outcomes and lower mortality rates (Moja et al. 2012, Vrahas and Sax 2017, Seong et al. 2020), we recommend early surgical intervention for patients with proximal femoral fractures who are confirmed positive for COVID-19 when symptoms of the illness are tolerable.

**Table:** 

Patient factors	Patient 1			Patient 2		
Age	81			83		
Sex	Female			Female		
BMI	29.5			17.9		
Lab findings	Initial	Pre-op.	Post-op.	Initial	Pre-op.	Post-op.
White-cell count (per mmі)	11.4K	11.6K	11.0K	9.1K	6.6K	7.2K
Hemoglobin (g/L)	9.2	8.7	10.1	13.7	11.8	8.4
Platelet (per mmі)	267K	297K	270K	141K	98K	94K
PT (sec)	16	17		15		
APTT (sec)	41	41		31		
Creatinine (µmol/L)	2.1	1.6	1.6	0.8	0.8	0.6
EGFR (mL/min/1.73mІ)	22	30	30	65	71	85
Albumin (g/L)	3.1	3.2		4		
CRP (mg/L)	163	85		37	106	109
Procalcitonin (ng/mL)	0.22	0.09		0.24		
D-dimer (mg/L)	3.8			14		

Abbreviations: PT, prothrombin time; APTT, activated partial thromboplastin time;

EGFR, estimated glomerular filtration rate; CRP, C-reactive protein.

## References

[CIT0001] Forrester J D, Nassar A K, Maggio P M, Hawn M T. Precautions for operating room team members during the COVID-19 pandemic. J Am Coll Surg 2020; Apr 2. pii: S1072-7515(20)30303-3. [Epub ahead of print] 10.1016/j.jamcollsurg.2020.03.030 PMC7270564 32247836

[CIT0002] Hong K, Lee S, Kim T, Huh H, Lee J, Kim S, Park J, Kim G, Sung H, Roh K. Guidelines for laboratory diagnosis of coronavirus disease 2019 (COVID-19) in Korea. Ann Lab Med 2020; 40(5): 351–60. 32237288 10.3343/alm.2020.40.5.351 PMC7169629

[CIT0003] Lin J, Ouyang J, Peng X-R, Isnard S, Fombuena B, Routy J-P, Chen Y-K. Potential therapeutic options for COVID-19: using knowledge of past outbreaks to guide future treatment 2020. Chin Med J 2020; March 17. 10.1097/CM9.0000000000000816 PMC7289304 32209887

[CIT0004] Mandernach M W, Beyth R J, Rajasekhar A. Apixaban for the prophylaxis and treatment of deep vein thrombosis and pulmonary embolism: an evidence-based review. Ther Clin Risk Manag 2015; 11: 1273–82. 26345156 10.2147/TCRM.S68010 PMC4556259

[CIT0005] Moja L, Piatti A, Pecoraro V, Ricci C, Virgili G, Salanti G, Germagnoli L, Liberati A, Banfi G. Timing matters in hip fracture surgery: patients operated within 48 hours have better outcomes. A meta-analysis and meta-regression of over 190,000 patients. PLoS One 2012; 7(10): e46175. 23056256 10.1371/journal.pone.0046175 PMC3463569

[CIT0006] Müller M E, Nazarian S, Koch P, Schatzker J. The comprehensive classification of fractures of long bones. Berlin: Springer-Verlag; 2012/1990.

[CIT0007] Olawin A M, Das J M. Spinal anesthesia. StatPearls [Internet]. Treasure Island, FL: StatPearls Publishing; 2019.

[CIT0008] Onder G, Rezza G, Brusaferro S. Case-fatality rate and characteristics of patients dying in relation to COVID-19 in Italy. JAMA 2020; Mar 23. [Epub ahead of print] 32203977 10.1001/jama.2020.4683

[CIT0009] Seong Y J, Shin W C, Moon N H, Suh K T. Timing of hip-fracture surgery in elderly patients: literature review and recommendations. Hip Pelvis 2020; 32(1): 11–16. 32158724 10.5371/hp.2020.32.1.11 PMC7054076

[CIT0010] Ti L K, Ang L S, Foong T W, Ng B S W. What we do when a COVID-19 patient needs an operation: operating room preparation and guidance. Can J Anaesth 2020: 1–3. 10.1007/s12630-020-01617-4 PMC7090746 32144591

[CIT0011] Vrahas M S, Sax H C. Timing of operations and outcomes for patients with hip fracture: it’s probably not worth the wait. JAMA 2017; 318(20): 1981–2. 29183051 10.1001/jama.2017.17624

[CIT0012] Wang C, Horby P W, Hayden F G, Gao G F. A novel coronavirus outbreak of global health concern. Lancet 2020; 395(10223): 470–3. 31986257 10.1016/S0140-6736(20)30185-9 PMC7135038

